# Introduction to the *Toxins* Special Issue on Dietary and Non-Dietary Phytochemicals and Cancer

**DOI:** 10.3390/toxins9010012

**Published:** 2016-12-28

**Authors:** Carmela Fimognari

**Affiliations:** Department for Life Quality Studies, Alma Mater Studiorum-University of Bologna, Corso d’Augusto 237, Rimini 47921, Italy; carmela.fimognari@unibo.it

The role of many phytochemicals in the modulation of the carcinogenesis process has been well documented by combining in vitro and animal studies, as well as epidemiological evidence. When acting in synergy, phytochemicals exert potential anti-cancer properties, and much progress has been made in defining their many biological activities at the molecular level. However, an interesting feature in the field of phytochemicals and cancer is the role of some phytochemicals in promoting cancer development. This Special Issue of *Toxins* aims to provide a comprehensive look at the contribution of dietary and non-dietary phytochemicals to cancer development and at the molecular mechanisms by which phytochemicals inhibit or promote cancer.

Cancer stem cells represent a small subset of tumor cells endowed with uncontrolled proliferative capacity and indefinite potential for self-renewal that drive tumorigenesis. Considering the potential of cancer stem cells in the initial development of cancer, resistance to therapy and metastasis, they have become a critical target for the identification and development of new approaches to fight cancer. Oh et al. present an overview of phytochemicals targeting signaling pathways involved in stemness maintenance and survival of cancer stem cells [1]. Some examples include cyclopamine from the corn lily, curcumin from turmeric, and piperine from black and long peppers, sulforaphane from cruciferous vegetables, the soy isoflavone genistein, and blueberry polyphenols. Lu et al. [2] report the effect of ovatodiolide—a macrocyclic diterpenoid compound isolated from *Anisomeles indica*—of blocking the self-renewal capability of breast cancer stem cells and downregulating the expression of stemness genes on human breast cancer stem cells.

The detailed review by Ismail et al. [3] focuses on ellagitannins, a class of phytochemicals widely investigated for their chemopreventive and anticancer activities. With the aim of delineating and predicting their actual clinical potential, the authors present the dietary sources, the pharmacokinetics, and the evidence on the chemopreventive efficacy and the anticancer activity of ellagitannins. The chemopreventive effects of ellagitannins are linked to their antioxidant and anti-inflammatory properties. Their anticancer activity is imputable to different mechanisms, including inhibition of pro-inflammatory pathway, cell-cycle arrest ability, and proapoptotic properties. The pleiotropic nature of the mechanisms behind the anticancer activity of ellegitannins is also demonstrated by their ability to block angiogenesis and inhibit endothelial cell growth. However, as the authors point out, orally administered ellagitannins are characterized by a limited bioavailability. Their widely recognized anticancer efficacy may lead to the adoption of different administration routes to overcome their low bioavailability, such as the intravenous route. However, their toxicological profile after intravenous administration is lacking.

This Special Issue also features several research papers reporting selective effects of phytochemicals against cancer cells, for which follow brief synopses.

The article by Farhan et al. [4] highlights mechanistic studies which provide insights into the cytotoxic and anticancer activity of catechins. Catechin, epicatechin, epigallocatechin, and epigallocatechin-3-gallate—the four major constituents of green tea—exert a prooxidant action through redox recycling of copper ions, and induce cellular DNA breakage in human peripheral lymphocytes. The presence of copper ions boosts their genotoxic effect. Catechins (especially epigallocatechin-3-gallate) have antiproliferative activity on breast cancer cells. Copper levels are much higher in cancer cells than in non-transformed cells. Thus, cancer cells would be more susceptible to redox cycling between copper ions and catechins to generate reactive oxygen species and thus DNA breakage. Accordingly, the authors demonstrate that normal breast epithelial cells are quite resistant to the treatment with catechins, but their culture in medium enriched with copper make them more susceptible to catechins’ antiproliferative effect. Taking the results presented in the paper into account, catechins exert their preferential prooxidant cytotoxic effect against cancer cells. Chen et al. [5] investigate the effect of tenuifolide B—derived from the stems of *Cinnamomum tenuifolium*—on viability, cycle progression, apoptosis, reactive oxygen species production, mitochondrial depolarization, and DNA damage in transformed and non-transformed oral cells. Tenuifolide B reduces the viability of cancer cells through the induction of apoptotic cell death. The production of reactive oxygen species and the induction of DNA damage are probably involved in its cytotoxic effect. Of note, the effects of tenuifolide B seem to be selective for cancer cells. Indeed, its effects on normal oral cells are much less pronounced than on cancer cells. Nguyen et al. [6] show that papaya leaves are a potential source of anticancer compounds. They actually contain flavonoids or flavonoid glycosides, particularly compounds from the kaempferol and quercetin families. Moreover, aqueous and ethanolic extracts of leaves reduce the survival of human oral squamous cell carcinoma cells. Interestingly, the two fractions with acidic pH are selectively cytotoxic towards the cancer cells, and the phenolic and flavonoid content positively correlates with the differential effect on cell viability.

However, it is worth noting that some phytochemicals can have an ambivalent character (especially in the possible context of high dose therapeutic applications), and favor cancer development. Some papers of this Special Issue deal with this important aspect.

Turrini et al. [7] provide new insights into the anticancer mechanisms of the root extract of *Withania somnifera*, a plant used in Indian traditional medicine. The article presents the cytotoxic and cytostatic effects of the extract on human leukemia cells and suggests the role of reactive oxygen species in its cytotoxic activity. Of note, *Withania* induces the expression of specific molecules such as calreticulin, Hsp-70, and Hsp-90 on cancer cells, thus boosting the immunogenic profile of tumor cells and stimulating the innate immune system response. In order to elaborate a preliminary risk/benefit profile, the authors analyze the genotoxicity of the extract through the quantification of histone H2A.X phosphorylation (γ-H2A.X), a biomarker of double-strand DNA breaks. The extract is found to be genotoxic. Bearing in mind that DNA damage plays a well-established role in cancer initiation and poses serious risks for human safety [8], the genotoxicity of *Withania* should be carefully examined for an accurate prediction of its risk–benefit profile.

Burgos-Morón et al. [9] examine the genotoxicity of caffeic acid and a commercial lyophilized coffee extract in cells deficient in the critical DNA repair protein Fanconi anemia D2 and demonstrate that this kind of cell is hypersensitive to the DNA damage induced by caffeic acid and coffee compared to non-deficient cells. These results suggest that coffee and caffeic acid may increase the risk of cancer, particularly in people with germline or sporadic mutations in the DNA repair protein Fanconi anemia D2. Taking into account that caffeic acid accumulates in the urinary bladder, the risk of bladder cancer development may be particularly high. The authors also discuss the key role that caffeic acid and other coffee constituents—such as chlorogenic acid and hydroquinone, endowed with an antioxidant activity at low concentrations and a pro-oxidant activity at higher concentrations—may have in cancer development.

An overview of the effects of some phytoestrogens on cancer progression is presented by Lee et al. [10]. Genistein, resveratrol, kaempferol, and 3,3′-diindolylmethane were extensively studied for their anticancer effects and as alternatives for hormone replacement therapy. In particular, they can inhibit the epithelial–mesenchymal transition, which plays a key role in cancer migration, invasion, and metastasis, and modulate the signaling pathways and the expression of epithelial–mesenchymal transition-related markers, such as TGF-β and PI3K/Akt/mTOR/NF-κB. Nevertheless, phytoestrogens like genistein and resveratrol can have a biphasic effect and lead to cancer cell growth at lower concentrations and to inhibition of cancer cell growth at higher concentrations.

Kakehashi et al. [11] explore the estrogenic effects in the mammary gland and uterus and the carcinogenetic activity of a diet containing *Pueraria mirifica* powder in female rats. To this end, they use different experimental strategies: *Pueraria* administered to ovariectomized animals at doses of 0.03%, 0.3%, and 3% in a phytoestrogen-low diet for 2 weeks; a 4 week application to non-operated rats at a dose of 3% after 7,12-dimethylbenz[a]anthracene cancer initiation; postpubertal administration of 0.3% to 5-week-old non-operated animals for 36 weeks following initiation of mammary and endometrial carcinogenesis with 7,12-dimethylbenz[a]anthracene and *N*-ethyl-*N*′-nitro-*N*-nitrosoguanidine, respectively. In the first experimental setting, *Pueraria* increased uterus weight; in the second one, *Pueraria* stimulated cell proliferation in the mammary gland; in the third experimental model, it boosted mammary adenocarcinoma incidence. These data raise very important questions on the safety of long-term exposure to phytoestrogens with regard to effects on the mammary gland and endometrium. Different products containing *Pueraria mirifica* are widely available in the USA and Japan. Despite the data on its positive health effects, including increasing hair growth, improving appetite, and providing relief for ailments like osteoporosis and even cancer, it evokes an estrogen-like effect that should be considered to better understand its risk–benefit profile. More research has to be performed to better define the relationship between the hazardous and chemopreventive effects of phytoestrogens.

I hope that this Special Issue will provide readers a better understanding of the mechanism of action of phytochemicals in modulating the carcinogenetic process. These aspects have advanced particularly far in recent years, and are extremely useful for the definition of efficient preventive or therapeutic strategies against cancer. I would also like to thank all authors contributing to this Special Issue in Toxins for their commitment and time, and our reviewers for their expert input and critical evaluation of the papers.
